# Cancer care at the end of life: system-wide expenditure in a national health service

**DOI:** 10.1007/s00520-025-09964-y

**Published:** 2025-10-21

**Authors:** Henriette Tind Hasse, Trine Kjær, Thea Otto Mattsson, Katrine Rahbek Schønnemann, Søren Rud Kristensen

**Affiliations:** 1 Danish Centre for Health Economics, Department of Public Health, University of Southern Denmark, Campusvej 55, Odense, 5230 Denmark; 2 Dept. of Oncology, Odense University Hospital, Odense, Denmark; 3 Dept. of Clinical Research, Odense University Hospital, University of Southern Denmark, Odense, Denmark

**Keywords:** Expenditure, Cancer care, End-of-life, Cancer, Healthcare, National health service

## Abstract

**Purpose:**

The provision of specialized palliative care (SPC) and the timely discontinuation of cancer-targeted treatments (CTT) are increasingly considered important in cancer care at the end of life (EoL). EoL cancer care decisions are often initiated in the hospital, and little is known about associated expenditure in other parts of the health system. Our primary objective was to examine the total healthcare and care setting-specific expenditure associated with either exposure to SPC or timely discontinuation of CTT for patients with cancer in the last 4 weeks of life. Our secondary objectives were to (1) examine how these expenditures evolved in the last 4 weeks of life and across care settings and (2) explore the relation between the associated expenditures of SPC and timely discontinuation of CTT. Exposure to SPC was defined by the first successful referral to SPC within the 6- to 1-month period (i.e., last 4 weeks) before death. Timely discontinuation of CTT was defined as receiving no CTT within the last 4 weeks of life.

**Methods:**

Using comprehensive linked Danish registry data, we conducted a nationwide matched cohort study (2011–2018), which analyzed care expenditure in various settings during the last 4 weeks of life for cancer patients, estimating costs with generalized linear model (GLM) and generalized estimating equation (GEE) models, and using logistic regression to assess SPC and timely discontinuation of CTT.

**Results:**

The total EoL care expenditure in the last 4 weeks of life was €3140 (96% CI €−3433 to €−2848) lower for patients exposed to SPC compared with non-exposed, mainly due to reduced hospital expenditure. Individuals exposed to timely discontinuation of CTT had €3430 (95% CI €−3649 to €−3211) lower expenditure per patient despite higher community, home-based, hospice, and primary care expenditure.

**Conclusion:**

Our findings show the development of EoL care expenditure during cancer patients’ final 4 weeks of life and can inform policymakers about the potential implications across the health system of changes in EoL care patterns.

**Supplementary information:**

The online version contains supplementary material available at 10.1007/s00520-025-09964-y.

## Introduction

There is an increasing emphasis on enhancing the quality of end-of-life (EoL) care for cancer patients, and integrated, high-quality EoL care is recognized as a critical component of comprehensive cancer treatment alongside the prevention and treatment of curable cancer [1]. This shift reflects a broader understanding of the need to prioritize the well-being of patients in their final stages of life. In 2018, the Lancet commission recommended an increased focus on the patient’s perspective and a more holistic approach to care with a full integration of specialized palliative care (SPC) into cancer-targeted and life-prolonging treatment to improve care, improve symptom management, and improve quality of life [2].

Cancer-targeted treatments (CTT) administered near the time of death have been associated with a range of negative outcomes, such as decreased quality of life, longer hospitalization, increased risk of serious adverse events, and even early death, whereas the evidence of positive effects on the underlying cancer disease is limited [3, 4]. In an everyday clinical setting, it may be difficult to determine whether a treatment is warranted due to prognostic uncertainty [5]. Previous studies highlight that a range of tools is available to assess the proximity to death and whether a patient will benefit from CTT [4, 6]. These tools include performance status, biomarkers, and clinical assessment [7, 8]. Moreover, the relationship between SPC and timely discontinuation of CTT may be complex and depend on the individual setting. Whereas some studies have found that SPC leads to a reduction in CTT, others report delayed entry into SPC due to CTT [9, 10].

Early initiation of SPC, combined with less intensive CTT, has been shown to reduce healthcare expenditure [11, 12]. This suggests that SPC has the dual potential to free up healthcare resources by limiting the use of aggressive treatment in the late stages of the disease and to reduce the number of days in hospital [13]. Most of these studies are single-site studies and focus on hospital expenditure in a US setting; however, little is known about the cost implications of introducing SPC outside the hospital and in a public health system with universal coverage [2, 14]. Decisions about care in one part of the health care system may have implications for other parts. Community and home-based care expenditure could be substantial and is important to consider when investigating the expenditure of cancer care. It has been documented that the majority of terminally ill patients wish to die at home [15], which often requires substantial resources in terms of community and home-based care services as well as primary care services [15].

One recent Danish study identified expenditures associated with both cancer and non-cancer deaths but did not consider the impact of care choices or community and home-based care expenditure [16]. Additionally, SPC is delivered in close collaboration between the specialist, the GP, and community and home-based care services, which may influence the setting-specific care expenditure [17].

Whereas it is obvious that the timely discontinuation of CTT will decrease direct hospital expenditure, the associated expenditure in other parts of the healthcare sector is unknown. Thus, research that examines the system-wide expenditure of patients receiving SPC and timely discontinuation of CTT is needed to support decision-making about how to deliver cost-effective high-quality care [18].

Our primary objective was to examine the total healthcare and care setting-specific expenditure associated with either exposure to SPC or timely discontinuation of CTT for patients with cancer in the last 4 weeks of life. Exposure to SPC was defined by the first successful referral to SPC within the 6- to 1-month period (i.e., last 4 weeks) before death. Timely discontinuation of CTT was defined as receiving no CTT within the last 4 weeks of life. Our secondary objectives were to (1) examine how these expenditures evolved in the last 4 weeks of life and across care settings and (2) explore the relation between the associated expenditures of SPC and the timely discontinuation of CTT.

## Methods

### Setting and cohort

This nationwide retrospective cohort study was conducted using linked individual-level data from multiple Danish registries. The cohort included all adults (18 +) with cancer from 2011 to 2018, identified via the National Patient Register (LPR) [19] using ICD-10 codes DC00*-DC96*. Individuals alive as of December 31, 2018, were excluded. The Danish Cause of Death Register (DCDR) [20] was linked to identify the cause of death. This excluded individuals who died from non-cancer causes. The cohort was validated by linking to the National Cancer Register (CAR) [21]. If the DCDR and CAR diagnoses differed, the CAR diagnosis was used. The registries were linked via unique Danish personal identification numbers. For a full list of the included registries, see Appendix Table A1.

#### Expenditure

We computed aggregated and weekly expenditures for the last 4 weeks of life. Healthcare expenditures were calculated retrospectively by measuring expenditures incurred during the 4-week period preceding death. Expenditure was divided into four categories depending on the care setting: hospital, hospice, primary, and community and home-based care. Total expenditure was computed as the sum of these.

All in- and outpatient hospital care activity was collected from the LPR and merged with the Danish Diagnosis Related Group (DRG) tariffs [22]. Hospice care expenditure was available in the LPR DRG register. The pricing is negotiated between the Region and the hospice [23]. Primary care expenditure was collected from the Danish National Health Service Register and comprised expenditure on GP care, physiotherapy, and chiropractic treatment. Community and home-based care activity was collected as prescribed hours from the Register of Elderly Documentation. The expenditure was calculated using the rates set by the Danish Medicine Council: €36.92 per hour for nurses and €32.76 per hour for nurse assistants [24, 25]. All expenditure data were adjusted to 2020 prices using the Danish Regions’ Salary and Price index and presented in euros [26].

#### SPC and timely discontinuation of CTT

CTT refers to all treatments targeting the cancer disease, e.g., chemotherapy, immunotherapy, targeted treatments, and radiation. Exposure to SPC was collected from the Danish Palliative Care Database [27] and included exposure to all types of SPC (hospital, hospice, and team-based). Only individuals registered with an SPC referral and a corresponding SPC start date, indicating a successful referral, were coded as exposed to SPC. All individuals referred within the last 4 weeks of life were excluded from the SPC analysis, as the analysis was focused particularly on this period. All individuals referred more than 180 days before the time of death were excluded from both the SPC and the CTT analyses [28, 29].

Information on timely discontinuation of CTT was obtained from the LPR. Patients were defined as being exposed to timely discontinuation of CTT if they had not received any cancer-targeted treatment within the last 4 weeks of life [11, 30].

#### Control variables

Socio-demographics measured at the time of diagnosis included age (from the population register), sex (binary), area of residence (region/municipality from the LPR), household income, education level (low, medium, high as per the International Standard Classification of Education (ISCED) system), co-habitation status (living alone or not), and number of children (under/over 18). Age was grouped into 18–45, 46–65, 66–85, and 85+.

Disease/health status included cancer type (grouped by ICD-10 codes), duration, and comorbidities, measured by the Charlson Comorbidity Index (CCI) using LPR data. The CCI score was calculated based on hospital contacts and first diagnosis (ICD-10) [31].

#### Coarsened exact matching

To address selection biases in SPC enrollment [28] and/or timely discontinuation of CTT [32], we used coarsened exact matching (CEM) [33] to match individuals exposed to SPC or timely discontinuation of CTT with similar unexposed controls. CEM is a statistical method used to reduce confounding in observational studies. It works by temporarily grouping (or “coarsening”) variables into broader categories, then matching treated and control individuals exactly within these groups to create more balanced comparison groups (also, see Appendix for further information).

### Statistical analysis

#### The association between treatment choices and expenditure

Differences in end-of-life (EoL) expenditure between patients exposed and not exposed to SPC and timely CTT discontinuation were estimated using a generalized linear model (GLM). This statistical framework extends linear regression by allowing for non-normal outcome distributions (e.g., binomial, Poisson) and linking the mean of the outcome to predictors through a specified link function. To analyze expenditure trends over time approaching EoL, we applied a generalized estimating equations (GEE) regression model. GEE is an extension of GLMs used for correlated or clustered data (e.g., repeated measures), which provides population-averaged estimates while accounting for within-group correlation structures. Family and log link were chosen based on Akaike’s information criterion test. For further details on the methods, see [34, 35]. The analyses were conducted for each care setting-specific expenditure variable. The results are presented as adjusted mean expenditure and average marginal effects per patient.

#### The association between SPC and timely discontinuation of CTT

A correlation between SPC and CTT has been described in previous literature [10]; therefore, we employed a multiple logistic regression analysis to examine the correlation between SPC and CTT, as the strength of this correlation may affect the main results.

#### Robustness analysis

The GLM and GEE are established methods for analyzing healthcare expenditure [36]. To test the robustness of our results to the choice of method, we repeated the analyses for aggregated expenditure analyses, applying winsorization at the 98th percentile [37]. Winsorization at the 98th percentile means adjusting extreme values in your data so that anything above the 98th percentile is set to the value at the 98th percentile. This approach reduces the impact of outliers while retaining all data points. Also, our CEM matching strategy resulted in some unmatched cases. To assess the sensitivity of our results to the chosen CEM strategy and the exclusion of individuals referred to SPC within the last 4 weeks of life, we performed seven iterations that gradually loosened the matching criteria.

All statistical analyses were conducted on Statistics Denmark using Stata 18 software.

## Results

We enrolled 89,109 cancer-deceased individuals in the final cohort (Table 1). Following the exclusion of individuals referred to SPC within the last 4 weeks of life and the implementation of the SPC CEM model, 52,670 individuals, 17,072 of whom were exposed to SPC, were included in the analysis. The CTT CEM model yielded 68,763 individuals, 52,061 of whom were exposed to timely discontinuation of CTT, for analysis.

**Table 1:** Descriptive statistics grouped by populations exposed and unexposed to SPC and timely discontinuation of CTT before and after performing coarsened exact matching

	SPC population before matching		SPC population after matching		Timely discontinuation of CTT population before matching		Timely discontinuation of CTT population after matching	
Characteristics	Exposed	Unexposed	Exposed	Unexposed	Exposed	Unexposed	Exposed	Unexposed
No. of patients	20,161	47,010	17,072	35,598	70,825	18,284	52,061	16,702
Exposed to SPC	/	/	/	/	34,388 (48)	7711 (42)	25,383 (48)	6948 (42)
Timely discontinuation of CTT	17,341 (86)	36,437 (78)	14,736 (86)	27,583 (78)	/	/	/	/
Mean SPC time before death (days) (SD)	79 (40)	/	78 (40)	/	46 (44)	36 (41)	45 (43)	36 (41)
Age (SD)	68 (12)	74 (11)	69 (11)	74 (10)	72 (11)	67 (11)	73 (10)	68 (11)
Sex								
Female	10,123 (50)	20,486 (44)	8404 (49)	15,601 (44)	33,144 (47)	7913 (43)	23,636 (45)	7177 (43)
Male	10,038 (50)	26,524 (56)	8668 (51)	19,997 (56)	37,681 (53)	10,371 (53	28,425 (55)	9525 (57)
Cancer type								
Lung	5476 (27)	12,400 (26)	5155 (30)	11,210 (32)	17,756 (25)	6204 (34)	15,955 (31)	5995 (36)
Central nervous system	829 (4)	1308 (3)	575 (3)	748 (2)	2498 (4)	368 (2)	1065 (2)	309 (2)
Colorectal	2494 (12)	5958 (13)	2127 (13)	4754 (13)	9642 (14)	1403 (8)	6552 (13)	1308 (8)
Gastroenterological, other	4119 (21)	7607 (16)	3671 (21)	6592 (19)	14,368 (20)	2069 (11)	10,817 (21)	1992 (12)
Genitourinary	1117 (5)	2753 (6)	823 (5)	1801 (5)	4236 (6)	921 (5)	2630 (5)	793 (5)
Gynecological	1127 (6)	1519 (3)	877 (5)	1202 (3)	3188 (5)	617 (4)	2153 (4)	571 (3)
Head and neck	767 (4)	1465 (3)	477 (3)	729 (2)	2323 (3)	529 (3)	1017 (2)	413 (2)
Hematological	439 (2)	4687 (10)	372 (2)	2166 (3)	3759 (5)	2213 (12)	2926 (5)	1910 (12)
Breast	987 (5)	2307 (5)	814 (5)	1659 (5)	3096 (4)	1174 (6)	2347 (4)	1044 (6)
Malignant melanoma	343 (2)	745 (2)	174 (1)	246 (1)	1096 (2)	435 (2)	404 (1)	272 (2)
Prostate	1116 (5)	2984 (6)	964 (6)	2350 (7)	3704 (5)	1260 (7)	3015 (6)	1163 (7)
Other	1347 (7)	3277 (7)	1043 (6)	2141 (6)	5159 (7)	1091 (6)	3180 (6)	932 (5)
Mean time (days) from diagnosis until death (SD)	520 (490)	399 (511)	511 (484)	387 (502)	437 (505)	400 (479)	433 (500)	393 (474)
Living alone	7911 (39)	21,875 (47)	6544 (38)	15,402 (43)	32,229 (46)	6179 (34)	21,129 (41)	5502 (33)
Household income								
Low	3941 (19)	12,083 (26)	3354 (20)	9104 (25)	16,991 (24)	3334 (18)	12,122 (23)	2996 (18)
Medium–low	4080 (20)	11,535 (24)	3555 (21)	8920 (25)	16,711 (24)	3614 (20)	12,427 (24)	3313 (20)
Medium–high	4606 (23)	10,466 (22)	3947 (23)	7755 (22)	15,955 (23)	4370 (24)	11,936 (23)	4037 (24)
High	5593 (28)	8776 (19)	4681 (27)	6897 (19)	14,920 (21)	5405 (30)	11,612 (23)	4943 (30)
Missing income	1941 (10)	4150 (9)	1535 (9)	2922 (8)	6248 (9)	1561 (9)	3964 (8)	1413 (8)
Children	16,855 (84)	37,279 (80)	14,874 (87)	31,098 (87)	57,125 (81)	15,390 (84)	46,304 (89)	14,487 (87)
Level of education								
Low	7608 (38)	21,356 (45)	6677 (39)	16,645 (47)	30,606 (43)	6793 (37)	23,051 (44)	6307 (38)
Medium	10,735 (53)	21,696 (46)	9503 (56)	17,560 (49)	34,123 (48)	9954 (54)	27,033 (52)	9408 (56)
High	875 (4)	1332 (3)	450 (3)	669 (2)	2337 (3)	775 (4)	1126 (2)	519 (3)
Missing	943 (5)	2626 (6)	442 (2)	724 (2)	3759 (5)	762 (4)	851 (2)	468 (3)
Region								
North Jutland	3048 (15)	3658 (8)	2305 (13)	2614 (7)	7980 (11)	1744 (9)	5090 (10)	1500 (9)
Central Jutland	4319 (21)	9964 (21)	3700 (22)	7695 (22)	15,707 (22)	3590 (20)	11,584 (22)	3322 (20)
Southern Denmark	4251 (21)	12,011 (25)	3713 (22)	9143 (26)	16,595 (23)	4167 (23)	12,449 (24)	3800 (23)
Capital	5143 (26)	13,457 (29)	4527 (26)	10,191 (29)	18,891 (27)	5245 (29)	14,505 (28)	4845 (29)
Zealand	3400 (17)	7920 (17)	2827 (17)	5955 (17)	11,652 (17)	3538 (19)	8433 (16)	3235 (19)
Charlson comorbidity score								
0	15,432 (77)	33,782 (72)	13,491 (79)	27,012 (76)	51,793 (73)	14,050 (77)	40,527 (78)	13,127 (79)
1	4275 (21)	12,461 (26)	3443 (20)	8421 (24)	17,730 (25)	3890 (21)	11,321 (21.6)	3429 (20)
More than 1	454 (2)	767 (2)	138 (1)	165 (1)	1302 (2)	344 (2)	213 (0.4)	146 (1)
Mean aggregated expenditure (SD)								
Hospital care	€5211 (7164)	€9387 (13,747)	€5145 (7070)	€9306 (13,412)	€6930 (10,702)	€12,097 (12,808)	€6992 (10,756)	€11,926 (12,541)
Hospice care	€996 (2173)	€5 (160)**	€990 (2167)	€4 (147)**	€659 (1809)	€510 (1563)	€663 (1814)	€505 (1565)
Community and home-based care	€1667 (4465)	€1567 (5395)	€1726 (4667)	€1487 (5184)	€848 (2907)	€373 (1230)	€982 (4261)	€623 (3903)
Primary care	€146 (167)	€171 (170)	€113 (151)	€66 (110)	150€ (164)	€101 (134)	€163 (170)	€116 (142)
Total	€8020 (8253)	€11,129 (14,193)	€7974 (8272)	€10,864 (13,846)	€8437 (10,598)	€13,081 (12,663)	€8800 (11,270)	€13,170 (12,877)

*Note*: Values are percentages of patients unless stated otherwise, **82 individuals had hospice expenditure with no referral to SPC in unmatched data, 60 had hospice expenditure and no SPC referral in matched data

For both SPC and timely CTT discontinuation, the unadjusted mean aggregated expenditures remained stable before and after matching. The SPC matching improved the balance, especially for gastroenterological cancer patients, children, medium education, and CCI. The average time from SPC referral to death was 78 days. In the matched groups, 86% of the SPC-exposed patients and 78% of the SPC-unexposed patients had timely CTT discontinuation.

In the CTT groups, the match reduced the imbalance between the exposed and unexposed groups, particularly for female patients with lung cancer (largest proportion of cancer), low and medium education, and CCI. Forty-eight per cent of those exposed and 42% of those unexposed to timely discontinuation of CTT were exposed to SPC.

### Specialized palliative care and expenditure

The adjusted regression analysis (Table 2) showed that for patients exposed to SPC, the predicted mean total expenditure per patient in the last 4 weeks of life was lower than for those unexposed to SPC, yielding a negative average marginal effect of €3140. This is even though the patients exposed to SPC had higher expenditure on hospice, community and home-based care, and primary care. For the full regression of estimated marginal effects, see Appendix, Table A2.

**Table 2:** Predicted mean expenditure (aggregated) and average marginal effects by SPC exposure

	**Total**	**Hospice**	**Hospital**	**Community and home-based**	**Primary**
Predicted expenditure					
Unexposed	€11,007*** (€10,784–€11,229)	€4*** (€2–€6)	€9555*** (€9323–€9786)	€1359*** (€1293–€1426)	€66.64*** (€65–€68)
Exposed	€7867*** (€7720–€8013)	€967*** (€935–€999)	€5170*** (€5025–€5314)	€1527*** (€1442–€1612)	€114*** (€111–€116)
Marginal effect of exposure to SPC	€−3140*** (€−3433 to €−2848)	€963*** (€930–€995)	€−4385*** (€−4672 to €−4097)	€168*** (€52–€284)	€47.05*** (€44–€50)
**Observations**	52,670	52,670	52,670	52,670	52,670

95% confidence interval (95% CI), ***p < 0.01, **p < 0.05, *p < 0.1. Results from GLM regression models controlling for age, sex, habitat status, education, household income, parental status, municipality and region of residence, cancer type, Charlson comorbidity, time from diagnosis to death, and year of death

**Fig. 1:**
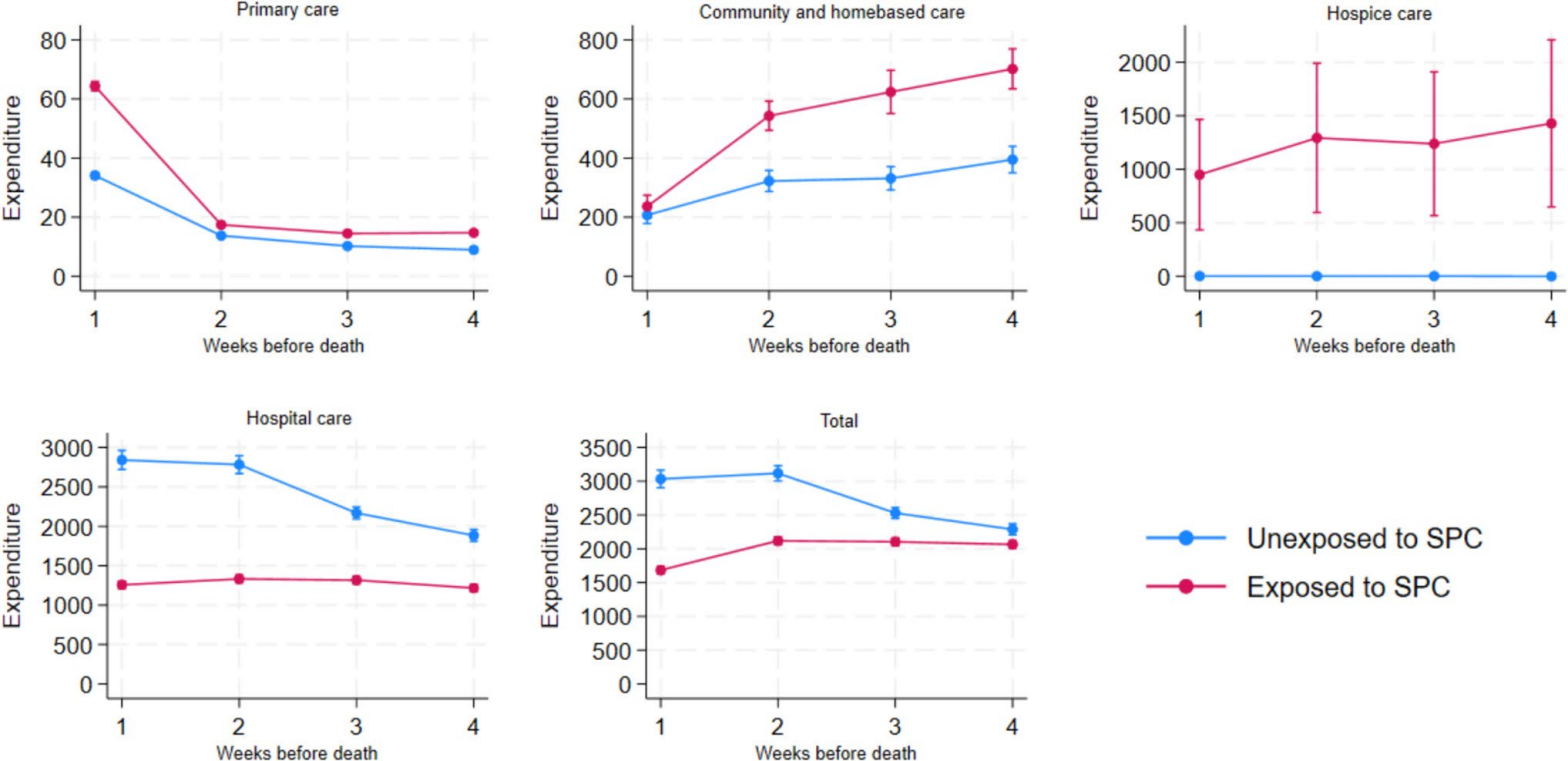
Weekly expenditure (Euro) by care setting and SPC exposure during the last 4 weeks of life  incl. 95% confidence intervals

The weekly expenditure results (Fig. 1) showed that the total mean expenditure (per patient) was lower for patients exposed to SPC in all 4 weeks prior to death. From the third week before death, the total expenditure in the unexposed group increased, whereas the total expenditure in the SPC-exposed group decreased from the second to the last week of life. In primary care, the patients exposed to SPC had slightly higher expenditure throughout the 4 weeks before death, but in the last week, the expenditure in the exposed group rose steeply compared to the unexposed group.

### Timely discontinuation of CTT and expenditure

The adjusted regression analysis (Table 3) showed that the predicted total mean expenditure was lower among patients exposed to a timely discontinuation of CTT than among their counterparts. In particular, hospital expenditure was lower in the SPC-exposed group with a negative marginal effect of €3911. The total expenditure in the CTT group was lower when compared to patients unexposed to timely discontinuation of CTT. The predicted hospice care expenditure in patients exposed to timely discontinuation of CTT was €168.8 higher than in patients who received CTT within the last 4 weeks of life. The expenditure associated with community and home-based and primary care was higher in the exposed group. For the full regression showing the estimated marginal effects of all variables included, see Appendix, Table A5.

**Table 3:** Predicted mean expenditure (aggregated) and average marginal effects according to timely discontinuation of CTT exposure

	**Total**	**Hospice**	**Hospital**	**Community and home-based**	**Primary**
Predicted expenditure					
Unexposed	€11,873*** (€11,670–€12,077)	€499.0*** (€475.1–€522.8)	€10,916*** (€10,690–€11,142)	€439.0*** (€411.5–€466.5)	€124.0*** (€121.8–€126.1)
Exposed	€8444*** (€8361–€8526)	€667.8*** (€648.8–€686.8)	€7005*** (€6921–€7089)	€589.5*** (€566.2–€612.8)	€163.1*** (€161.5–€164.7)
Marginal effect of exposure to timely discontinuation of CTT	€−3430*** (€−3649 to €−3211)	€168.8*** (€139.5–€198.2)	€−3911*** (€−4150 to €−3672)	€150.5*** (€120.1–€181.0)	€39.12*** (€36.51–€41.72)
Observations	68,763	68,763	68,763	68,763	68,763

95% confidence interval (95% CI), ***p < 0.01, **p < 0.05, *p < 0.1. Results from GLM regression models controlling for age, sex, habitat status, education, household income, parental status, municipality and region of residence, cancer type, Charlson comorbidity, time from diagnosis to death, and year of death

**Fig. 2:**
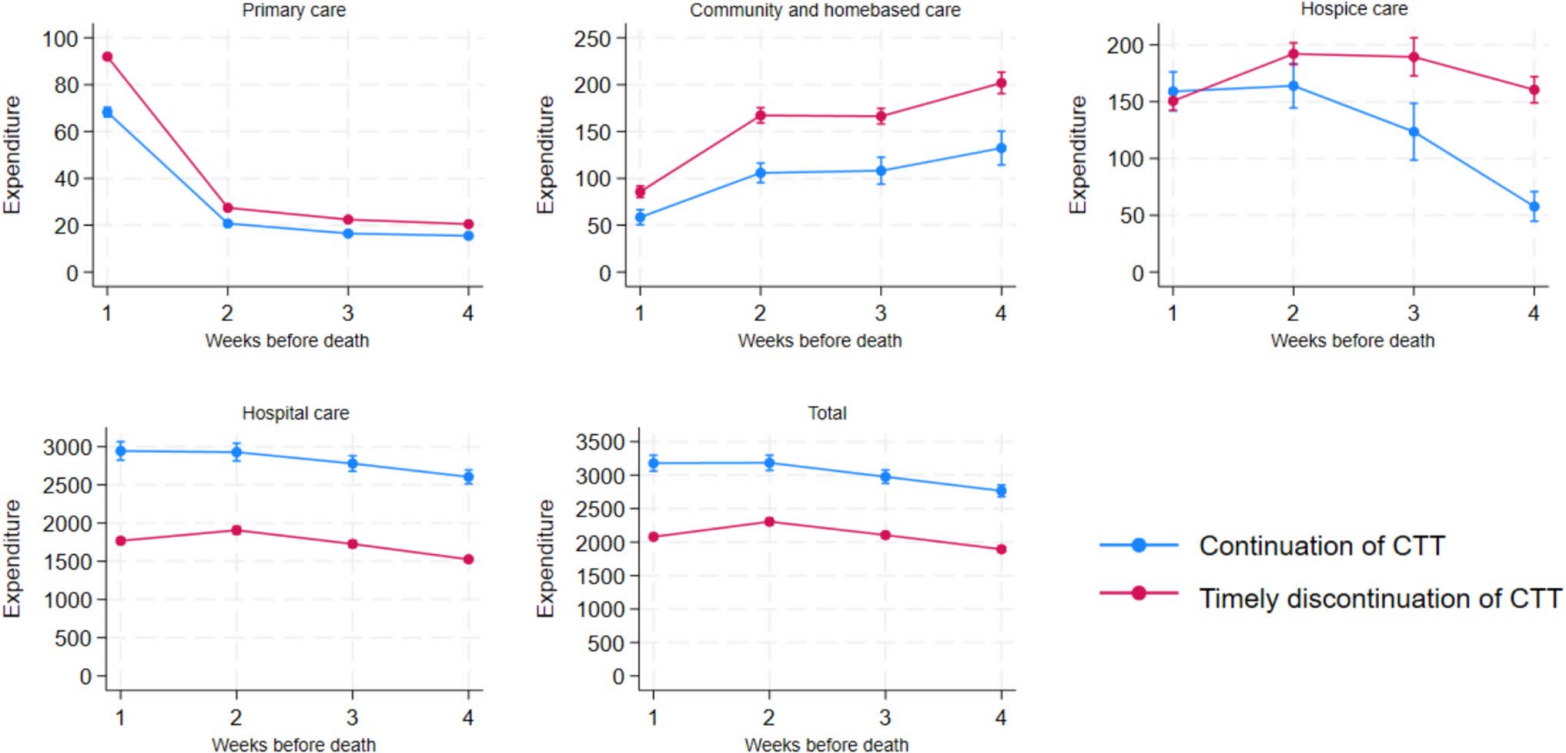
Weekly expenditure (euro) by care setting and timely discontinuation of CTT exposure during  the last 4 weeks of life incl. 95% confidence intervals

As presented in Fig. 2, the weekly total and hospital expenditure were lower throughout the 4 weeks prior to death for patients exposed to timely discontinuation of CTT when compared with patients that were unexposed. Primary, community, and home-based care expenditures were higher for patients exposed to timely discontinuation of CTT all 4 weeks. For hospice care, the curves intersected in the last week of life. In weeks 3 and 4, patients exposed to timely discontinuation of CTT had higher expenditure due to hospice care.

### Association between being exposed to SPC and timely discontinuation of CTT

The logistic regression analysis showed that there was a statistically significant association between being exposed to SPC and the timely discontinuation of CTT. The probability of timely discontinuation of CTT was OR 1.5 (CI 1.40548–1.5129) for individuals exposed to SPC.

### Robustness analysis

When winsorization was applied (Appendix, Table A3 and A4), the results remained in the same direction. Across all CEM iterations and for both SPC and CTT, the findings aligned consistently with the main analysis. See Appendix, Table A6 for the number of unmatched individuals by each iteration. For the aggregated expenditure results by each CEM iteration, see Appendix, Tables A7 (SPC) and A8 (CTT).

## Discussion

Our study provides new insight into the composition of cancer care expenditure in the last 4 weeks across treatment pathways. Lower hospital expenditure near the EoL for patients exposed to SPC compared with those unexposed to SPC aligns with the findings in previous studies [14, 28]. In addition, the lower total expenditure for patients exposed to SPC when compared with patients unexposed to SPC was not offset by higher community, home-based, and primary care expenditure or the utilization of hospice care. The expenditure in relation to SPC treatment was not isolated in the current study, but all expenditures were included in the DRGs and hospice expenditures [38]. SPC has previously been found to be associated with better symptom control, treatment satisfaction, no reduction in overall survival, and ultimately a better quality of life [39], but as we did not have information on healthcare gains (better quality of life, less pain, etc.) from SPC, we cannot claim that our results indicate that SPC is a cost-effective treatment choice when caring for cancer patients at the EoL. Previous studies have highlighted that there may be additional benefits to the patient and larger cost reductions by introducing SPC early (more than 90 days prior to death) [40]. In the current study, individuals referred prior to 180 days before death were excluded. Consequently, our estimated expenditure in the hospital sector may be overestimated, as we do not account for the additional benefits that this group may experience that could lead to further expenditure reductions in the hospital sector. As mentioned, these benefits could include better symptom control, a reduction in CTT, and thereby a reduction in potential adverse events, all of which potentially lead to fewer hospital admissions. Our estimated expenditure due to hospice may be underestimated, as an early referral to SPC may also have given access to hospice care earlier in the course of the disease. An earlier entry to SPC combined with cancer-targeted treatments would also be in line with the Lancet Commission recommendations [2]. In our study, the average time from SPC referral until death is 78 days, not including referrals during the last 4 weeks of life.

Our finding of lower expenditure in the group exposed to a timely discontinuation of CTT is in line with previous findings [40]. There may be many warranted reasons for administering CTT to patients until very late in their disease trajectory [41]. These reasons include patient wishes, societal pressure, and prognostic uncertainty. However, there is a lack of evidence of an effect on the underlying cancer disease when CTT is administered near the EoL [42, 43]; moreover, it has been well documented that CTT administered at the EoL increases the risk of severe adverse events resulting in hospital admittance [44]. Future research should investigate if lower hospital expenditure among patients exposed to timely discontinuation of CTT could be the result not only of lower direct treatment expenditure but also of fewer hospital admissions due to adverse events. Moreover, it would be relevant to investigate how patients are affected. For example, investigating whether quality of life is affected positively or negatively for the patient group with reduced expenditure at EoL could indicate whether the reduced expenditure is positive not only for society as a whole but also for the individual patient.

Both for patients exposed to SPC and for patients exposed to a timely discontinuation of CTT, the aggregated community and home-based care expenditure was higher and hospital expenditure was lower. Our findings align with prior research that showed a reciprocal relationship between care settings at the EoL. Hence, an increase in care within one setting corresponds to decreased care in others [45]. In our study, although higher expenditure on community and home-based care was observed, it was not offset by a reduction in hospital expenditure for patients exposed to SPC or timely discontinuation of CTT. In addition, the higher community and home-based care expenditure in both analyses may indicate that the patients spend more time at home. In previous literature, the number of days spent at home within the last 6 months of life has been presented as a patient-experienced quality indicator of care [46].

Our results show a positive association between being exposed to SPC and not receiving CTT within the last 4 weeks of life, which is in line with previous findings [10]. Moreover, individuals who were exposed to timely discontinuation of CTT utilize more hospice care than those not exposed. However, there was not a large difference in the proportions in the matched CTT groups, in which 42% of the individuals exposed and 48% of the patients unexposed to a timely discontinuation of CTT were exposed to SPC. Our results do not allow us to conclude the direction of the association. In all five analyses, the patients exposed and unexposed to a timely discontinuation of CTT had statistically significantly different expenditures throughout the analysis period, which may indicate that the groups were different when entering the time of analysis. This suggests that other factors may contribute to the difference in expenditure. In the SPC analysis, the primary care expenditures were similar until the last week of life, and the total expenditures were closer when entering the analysis period for the exposed and unexposed groups, increasing in difference when approaching the time of death.

### Strengths and limitations

The ability to combine data from several Danish registries, which are renowned for their completeness and validity [21], was a major strength in our study. To reduce the bias in selection, we applied CEM, which is a causal inference technique [47]. However, despite our efforts to conduct the best possible match, our empirical strategy only allowed us to measure associations. We cannot rule out that unobserved characteristics related to, e.g., lifestyle, disease progression, and especially patient needs could influence the magnitude of the estimated associations. Therefore, further analysis is needed to determine whether these effects are causal.

The treatment choices (SPC and timely discontinuation of CTT) included in this study have previously been highlighted as indicators of good clinical quality [48]. Whereas both measures have been found to have a positive effect on patients’ quality of life [15], we cannot assess the cost-effectiveness of the chosen treatment pathways, as patient preferences and/or needs near EoL could warrant both the absence of an SPC referral and the administration of CTT near the time of death.

We only included healthcare sector (including community and home-based care) expenditure; additional expenditure to the patient and their family in terms of informal care, lost earnings, and prescription drugs would be relevant to include in future research to take a wider societal perspective [16]. Including this expenditure may affect the relative distribution of the setting-specific expenditure associated with different treatment choices. Finally, we do not have information about patient-reported outcomes and therefore cannot investigate the association between expenditure and individual differences in realized (subjective) quality of life from the treatment.

## Conclusion

This nationwide retrospective cohort study demonstrates that the end-of-life care pathways of patients with cancer are associated with different healthcare expenditure patterns during the final 4 weeks of life. Patients receiving SPC had significantly lower total expenditure per patient, which was driven primarily by reduced hospital expenditure that was only partially offset by increased hospice, community, and primary care expenditure. Patients with timely discontinuation of CTT also had lower total expenditure, again mainly due to reduced hospital costs despite higher expenditure in other care settings.

The findings suggest that both SPC and timely discontinuation of CTT can facilitate a shift from the hospital setting to other settings and that such a shift is associated with lower total health expenditure. Although our matching strategy cannot establish causality, our results should be of interest to policymakers seeking to improve end-of-life care. Further, they highlight the potential resource implications of end-of-life care decisions across the healthcare system. Shifting palliative care from hospital to other settings and facilitating timely treatment discontinuation could lead to more efficient use of scarce healthcare resources but requires adequate out-of-hospital capacity.

## Supplementary information

Below is the link to the electronic supplementary material.

Supplementary file 1 (DOCX 68.8 KB)

## Data Availability

All data used in the analysis can be accessed through Statistics Denmark and the Danish Health Data Authority.
