# Supplementary material for: Cancer care at the end of life: system-wide expenditure in a national health service
Source: Support Care Cancer. 2025 Oct 21;33(11):972. doi: 10.1007/s00520-025-09964-y (PMC12540515; doi:10.1007/s00520-025-09964-y)
Supplement: Supplementary file 1 — (DOCX 68.8 KB) [file 520_2025_9964_MOESM1_ESM.docx]

# Appendix

**Table A1.** Table of all included data sources

| Danish register | Variables |
| --- | --- |
| The Population Statistics Register (BEF and FAIN) | Age, sex, citizenship, immigration, address, parents, partners, children |
| The Cancer Register (CAR) | Information on type of cancer |
| The Register of Causes of Death (DODSAARS and DODSAASG) | Time, cause, and manner of death |
| The Danish Register for Evaluation of Marginalization (DREAM) | Sector of employment, public transfers, emigration, death |
| The National Patient Register (LPR)  (DRGSOMA_AMB, DRGSOMA_HEL, DRGSOMA_KONTAKT, LPR_ADM, LPR_BES, LPR_DIAG, PRIV_ADM, PRIV_BES, UAF_ADM, UAF_BES, UAF_DAIG) | Information on treatment at somatic public or private hospitals, admission and discharge, diagnosis, examination, treatment, accidents, waiting time, tariffs |
| The Family Income Statistics Register (FAIK) | Family income, wealth, taxes, public transfer payments, private pension contributions and payouts, housing |
| The Income Statistics Register (IND) | Income, wealth, taxes, public transfer payments, private pension contributions and payouts, housing |
| The Danish National Health Service Register (SSSY and SYSI) | Contacts with, e.g., general practice, dentists, physiotherapists, and psychologists, dates and charges |
| The Population's Education Register (UDDA) | Ongoing and highest completed education |
| RKKP registers and databases |  |
| The Danish Palliative Database | Reference status, point of reference, place of first visit, and reason for referral decline. |

**Table A2**. Full regression of average marginal effects according to SPC exposure

|  | Total | Hospice | Hospital | Community and home-based | Primary |
| --- | --- | --- | --- | --- | --- |
|  |  |  |  |  |  |
| Exposed to SPC | €-3,140*** | €-4,385*** | €962.5*** | €167.7*** | €47.05*** |
|  | (€-3,433 – €-2,848) | (€-4,672 – €-4,097) | (€930.1 – €994.8) | (€51.79 – €283.7) | (€44.01 – €50.09) |
| Female | -494.9** | -546.0** | 26.01** | 64.96 | -8.394*** |
|  | (-886.3 – -103.4) | (-970.9 – -121.1) | (0.212 – 51.80) | (-67.91 – 197.8) | (-11.45 – -5.341) |
| Region of residence (ref. North Jutland) | | | | | |
| Central Jutland | -1,174*** | -1,277*** | 57.93*** | 515.8*** | 14.97*** |
|  | (-1,954 – -394.5) | (-2,156 – -398.7) | (23.05 – 92.81) | (299.4 – 732.3) | (10.28 – 19.67) |
| Southern Denmark | -1,726*** | -1,890*** | 41.13 | 341.4** | 25.97*** |
|  | (-2,852 – -600.9) | (-3,145 – -634.5) | (-9.883 – 92.14) | (11.37 – 671.4) | (18.43 – 33.52) |
| Capital | -1,608* | -1,642 | 189.0*** | -27.48 | -29.09*** |
|  | (-3,479 – 263.6) | (-3,645 – 360.8) | (69.38 – 308.6) | (-600.0 – 545.1) | (-40.90 – -17.28) |
| Zealand | -1,425* | 74.59 | -1,514* | 194.4 | -2.881 |
|  | (-2,978 – 128.3) | (-58.65 – 207.8) | (-3,262 – 233.7) | (-253.7 – 642.5) | (-18.17 – 12.41) |
| Year of death (ref. 2011) | | | | | |
| 2012 | -94.24 | 168.4 | 47.16* | 58.85 | 0.538 |
|  | (-892.7 – 704.3) | (-727.7 – 1,065) | (-1.180 – 95.50) | (-37.35 – 155.1) | (-5.346 – 6.421) |
| 2013 | 895.8* | 1,091** | 55.67** | 155.0*** | 12.61*** |
|  | (-13.83 - 1,805) | (233.2 - 1,950) | (6.563 - 104.8) | (46.76 - 263.3) | (6.585 - 18.64) |
| 2014 | 1,008*** | 1,223*** | 94.35*** | 233.0*** | 13.03*** |
|  | (268.5 – 1,747) | (443.8 – 2,002) | (40.64 – 148.1) | (119.8 – 346.3) | (6.873 – 19.20) |
| 2015 | 365.1 | 630.2 | 93.81*** | 473.7*** | 13.50*** |
|  | (-424.7 – 1,155) | (-227.2 – 1,488) | (41.39 – 146.2) | (338.2 – 609.2) | (7.287 – 19.72) |
| 2016 | 1,005*** | 180.0 | 78.10*** | 1,773*** | 0.0741 |
|  | (266.3 – 1,743) | (-615.5 – 975.6) | (27.02 – 129.2) | (1,603 – 1,944) | (-6.040 – 6.188) |
| 2017 | 2,306*** | 564.6 | 101.6*** | 2,484*** | -5.500* |
|  | (1,496 - 3,115) | (-303.9 - 1,433) | (47.42 - 155.8) | (2,258 - 2,709) | (-11.45 - 0.453) |
| 2018 | 1,557*** | -375.4 | 181.0*** | 2,690*** | 5.697* |
|  | (841.5 – 2,272) | (-1,100 – 348.8) | (125.7 – 236.4) | (2,484 – 2,895) | (-0.843 – 12.24) |
| Education (ref. low) |  |  |  |  |  |
| Medium | 253.0 | 378.3** | 44.07*** | -92.04 | -2.521* |
|  | (-86.18 – 592.2) | (29.57 – 727.1) | (18.23 – 69.92) | (-222.2 – 38.11) | (-5.474 – 0.432) |
| High | 23.87 | -21.16 | 66.00* | -341.4** | -4.023 |
|  | (-1,064 – 1,112) | (-1,219 – 1,177) | (-2.995 – 135.0) | (-645.0 – -37.77) | (-14.35 – 6.303) |
| Missing | 1,941 | 1,978 | -53.53 | 457.3* | -3.950 |
|  | (-1,443 – 5,325) | (-1,665 – 5,621) | (-120.6 – 13.57) | (-23.72 – 938.2) | (-13.08 – 5.178) |
| Living alone | -591.4*** | -1,302*** | 26.11* | 728.2*** | -14.96*** |
|  | (-947.7 – -235.1) | (-1,663 – -942.1) | (-1.168 – 53.39) | (575.0 – 881.3) | (-17.68 – -12.23) |
| Age group (ref. 18–45) |  |  |  |  |  |
| 46–65 | -3,921*** | -4,903** | -31.91 | 461.5*** | 20.33*** |
|  | (-6,825 – -1,017) | (-8,747 – -1,060) | (-103.8 – 39.98) | (256.0 – 667.0) | (11.55 – 29.12) |
| 66–85 | -5,496*** | -6,918*** | -59.50 | 1,095*** | 21.85*** |
|  | (-8,358 – -2,634) | (-10,674 – -3,161) | (-131.4 – 12.36) | (900.1 – 1,290) | (13.32 – 30.38) |
| 86–106 | -7,597*** | -9,793*** | -99.92** | 1,811*** | 15.03*** |
|  | (-10,479 – -4,715) | (-13,516 – -6,070) | (-191.1 – -8.765) | (1,522 – 2,100) | (5.272 – 24.80) |
| Cancer type (ref. lung cancer) | | | | | |
| Central nervous system | -833.7* | -1,339*** | 31.67 | 861.3*** | 14.80*** |
|  | (-1,757 – 89.74) | (-2,246 – -432.7) | (-38.64 – 102.0) | (456.1 – 1,266) | (4.093 – 25.51) |
| Colorectal | 1,429*** | 1,392*** | 8.159 | 175.2** | -5.113** |
|  | (736.9 – 2,122) | (573.3 – 2,212) | (-30.01 – 46.32) | (5.557 – 344.9) | (-9.492 – -0.735) |
| Gastroenterological | -46.43 | -55.90 | 50.60*** | -130.4* | -0.680 |
|  | (-640.5 – 547.6) | (-703.1 – 591.3) | (17.94 – 83.26) | (-281.6 – 20.85) | (-4.183 -– 2.823) |
| Genitourinary | 548.1 | 271.5 | 22.49 | 188.0 | 11.90*** |
|  | (-233.6 – 1,330) | (-515.2 – 1,058) | (-35.84 – 80.83) | (-54.10 – 430.2) | (5.433 – 18.37) |
| Gynecological | 185.5 | -66.05 | 86.66*** | 258.8 | 1.714 |
|  | (-810.6 – 1,182) | (-1,112 – 979.5) | (27.82 – 145.5) | (-105.1 – 622.6) | (-5.942 – 9.371) |
| Head and neck | 1,433* | 1,345 | 22.80 | 694.2*** | -6.531* |
|  | (-46.26 – 2,912) | (-339.1 – 3,029) | (-48.59 – 94.19) | (240.1 – 1,148) | (-13.70 – 0.635) |
| Hematology | 6,743*** | 7,066*** | -5.624 | 70.65 | -8.067** |
|  | (5,536 – 7,951) | (5,748 – 8,384) | (-88.61 - 77.36) | (-223.6 – 364.9) | (-15.06 – -1.078) |
| Breast | -17.12 | -260.1 | -49.24** | 136.7 | 5.243 |
|  | (-827.4 – 793.2) | (-1,172 – 652.0) | (-96.66 – -1.823) | (-105.2 – 378.5) | (-2.467 – 12.95) |
| Malignant melanoma | -1,416*** | -1,312*** | 18.91 | 334.5 | -0.201 |
|  | (-2,349 – -482.5) | (-2,222 – -402.5) | (-101.0 – 138.8) | (-347.4 – 1,016) | (-12.84 – 12.44) |
| Other | 671.0* | 637.2 | -42.97* | 81.76 | 3.591 |
|  | (-86.67 – 1,429) | (-162.5 – 1,437) | (-87.85 – 1.919) | (-217.6 – 381.1) | (-2.356 – 9.538) |
| Prostate | 81.22 | -515.6 | -12.97 | 411.3*** | 13.99*** |
|  | (-523.5 – 685.9) | (-1,146 – 114.8) | (-71.17 – 45.23) | (140.8 – 681.9) | (7.794 – 20.18) |
| Household income (ref. low) | | | | | |
| Medium-low | 178.1 | 134.5 | 35.78** | -57.73 | 1.101 |
|  | (-327.3 – 683.4) | (-423.0 – 692.0) | (0.598 – 70.95) | (-214.1 – 98.61) | (-2.672 – 4.874) |
| Medium-high | 266.7 | 336.7 | 63.46*** | -151.6* | 1.861 |
|  | (-470.5 – 1,004) | (-487.7 – 1,161) | (27.92 – 99.00) | (-328.3 – 25.13) | (-2.092 – 5.814) |
| High | 734.3*** | 868.4*** | 78.23*** | -322.5*** | -0.652 |
|  | (183.8 – 1,285) | (284.3 – 1,452) | (40.94 – 115.5) | (-524.6 – -120.3) | (-5.039 – 3.734) |
| Missing household income | -799.5** | -176.6 | 82.89*** | -413.2*** | 5.007 |
|  | (-1,452 – -146.5) | (-893.2 – 540.0) | (21.66 – 144.1) | (-717.3 – -109.1) | (-1.248 – 11.26) |
| Children (ref. no children) | | | | | |
| Has child under 18 | -1,007 | -486.6 | 100.4 | 305.0 | 3.434 |
|  | (-4,942 – 2,928) | (-4,386 – 3,413) | (-199.4 – 400.2) | (-2,057 – 2,667) | (-28.45 – 35.32) |
| Has child over 18 | 817.3*** | 794.6*** | 24.35 | 24.77 | 9.890*** |
|  | (271.0 – 1,364) | (195.7 – 1,393) | (-11.25 – 59.95) | (-141.2 – 190.8) | (5.792 – 13.99) |
| Charlson Comorbidity | 156.6 | 78.88 | -7.955 | 34.00 | 1.070 |
|  | (-45.55 – 358.8) | (-153.0 – 310.7) | (-19.79 – 3.879) | (-18.52 86.52) | (-0.222 – 2.362) |
| Time from death diagnosis until death | -1.781*** | -3.660*** | 0.00843 | 0.503*** | 0.000635 |
|  | (-2.474 – -1.088) | (-4.887 – -2.433) | (-0.0196 –0.0364) | (0.401 – 0.606) | (-0.00293 – 0.00420) |
| Observations | 52,670 | 52,670 | 52,670 | 52,670 | 52,670 |
| Note: 95% confidence interval (95% CI), *** p<0.01, ** p<0.05, * p<0.1. Municipality of residence not displayed. | | | | | |

10.1. Winsorization

In the SPC analysis, the winsorized total expenditure was € 1,365 (95% CI €-1,509 to €-1,221) lower, and the hospital expenditure was €2,744 (95% CI €-2,867 to €-2,622) lower in the SPC-exposed group when compared with the unexposed group. Community and home-based care expenditure for patients exposed to SPC was €298.9 higher when compared with those unexposed to SPC. The per-patient expenditure due to hospice and primary care remained relatively similar to that estimated in the main analysis in both the group exposed to SPC and the unexposed group.

The estimated winsorized expenditure for the analysis of timely discontinuation of CTT was very similar to the main analysis.

**Table A3**. Average marginal effects according to SPC exposure (Winsorized)

|  | Total | Hospital | Hospice | Community and home-based | Primary |
| --- | --- | --- | --- | --- | --- |
| Marginal effect of exposure to SPC | €-1,365*** | €-2,744*** | €928.7*** | €298.9*** | €40.65*** |
|  | (€-1,509 – €-1,221) | (€-2,867 – €-2,622) | (€898.9 – €958.6) | (€199.0 – €398.7) | (€37.85 – €43.44) |
| Observations | 52,670 | 52,670 | 52,670 | 52,670 | 52,670 |
| Note: 95% confidence interval (95% CI), *** p<0.01, ** p<0.05, * p<0.1. | | | | | |

**Table A4**. Average marginal effects according to exposure to timely discontinuation of CTT (Winsorized)

|  | Total | Hospice | Hospital | Community and home-based | Primary |
| --- | --- | --- | --- | --- | --- |
| Marginal effect of exposure to timely discontinuation of CTT | €-3,432*** | €165.8*** | €-3,798*** | €93.01*** | €39.12*** |
|  | (€-3,566 – €-3,299) | (€138.4 –€193.2) | (€-3,946 – €-3,650) | (€73.08 –€ 112.9) | (€36.51 – €41.72) |
| Observations | 68,763 | 68,763 | 68,763 | 68,763 | 68,763 |
| Note: 95% confidence interval (95% CI), *** p<0.01, ** p<0.05, * p<0.1. | | | | | |

**Table A5**. Full regression of average marginal effects according to exposure to timely discontinuation of CTT

|  | Total | Hospice | Hospital | Community and home-based | Primary |
| --- | --- | --- | --- | --- | --- |
|  |  |  |  |  |  |
| Timely discontinuation of CTT | €-3,418*** | €198.1*** | €-3,917*** | €152.6*** | €39.26*** |
|  | (€-3,636 – €-3,201) | (€158.8 – €237.4) | (€-4,155 – €-3,678) | (€119.9 –€185.3) | (€36.67 –€41.84) |
| Female | -725.2*** | 182.6*** | -917.8*** | 15.85 | -5.345*** |
|  | (-902.2 – -548.2) | (136.6 –228.6) | (-1,101 – -735.1) | (-20.49 – 52.20) | (-8.248 – -2.441) |
| Region of residence (ref. North Jutland) | | | | | |
| Central Jutland | 717.6 | 351.2 | 860.3 | 20.93 | -28.03 |
|  | (-2,451 – 3,887) | (-361.2 –1,064) | (-2,482 – 4,203) | (-584.1 – 626.0) | (-96.98 – 40.93) |
| Southern Denmark | 893.2 | -65.04 | 1,084 | 153.2 | -40.52 |
|  | (-2,324 – 4,110) | (-665.3 – 535.3) | (-2,353 – 4,520) | (-542.4 – 848.7) | (-110.5 – 29.48) |
| Capital | 778.2 | 235.6 | 358.3 | 312.1 | -51.27 |
|  | (-2,377 – 3,933) | (-476.8 – 948.1) | (-2,863 – 3,580) | (-468.7 – 1,093) | (-118.8 – 16.30) |
| Zealand | 1,528 | -140.5 | 694.4 | 302.4 | -44.37 |
|  | (-1,774 – 4,830) | (-769.3 – 488.2) | (-2,615 – 4,004) | (-478.8 – 1,084) | (-114.5 – 25.72) |
| Year of death (ref. 2011) | | | | | |
| 2012 | -208.8 | -60.12 | -126.1 | 38.44*** | -0.233 |
|  | (-549.6 - 132.0) | (-140.5 - 20.22) | (-495.0 - 242.7) | (19.41 - 57.46) | (-6.123 - 5.656) |
| 2013 | 434.0** | -29.43 | 500.7** | 36.51*** | 13.83*** |
|  | (80.16 – 787.9) | (-113.3 – 54.47) | (117.3 – 884.1) | (17.23 – 55.78) | (7.647 – 20.01) |
| 2014 | 778.3*** | 124.9*** | 719.9*** | 73.06*** | 5.153* |
|  | (420.6 – 1,136) | (32.07 – 217.8) | (334.0 – 1,106) | (51.52 – 94.60) | (-0.930 – 11.24) |
| 2015 | 132.0 | 68.39 | -21.45 | 174.1*** | 9.425*** |
|  | (-220.0 – 484.0) | (-21.46 – 158.2) | (-398.0 – 355.1) | (145.7 – 202.5) | (3.225 – 15.63) |
| 2016 | 390.6** | 69.16 | -285.5 | 590.7*** | -14.03*** |
|  | (30.81 – 750.3) | (-21.62 – 159.9) | (-662.0 – 91.09) | (532.4 – 649.0) | (-19.96 – -8.100) |
| 2017 | 836.2*** | 56.35 | -168.9 | 1,060*** | -16.36*** |
|  | (466.5 – 1,206) | (-33.53 – 146.2) | (-550.4 – 212.5) | (965.5 – 1,154) | (-22.30 – -10.41) |
| 2018 | -43.04 | 125.7*** | -1,144*** | 1,204*** | -2.606 |
|  | (-406.1 – 320.1) | (30.18 – 221.1) | (-1,515 – -772.6) | (1,095 – 1,313) | (-8.806 – 3.594) |
| Education (ref. low) |  |  |  |  |  |
| Medium | 146.9* | 111.2*** | 79.28 | -39.06** | -8.053*** |
|  | (-25.17 – 318.9) | (69.57 – 152.9) | (-99.43 – 258.0) | (-74.21 – -3.905) | (-10.85 – -5.259) |
| High | 31.39 | 311.0*** | -207.4 | 7.710 | -10.78** |
|  | (-538.6 – 601.4) | (118.6 – 503.4) | (-784.4 – 369.6) | (-117.3 – 132.8) | (-19.72 – -1.837) |
| Missing | 140.0 | -90.55 | 141.3 | 30.82 | -1.873 |
|  | (-488.1 – 768.1) | (-208.4 – 27.34) | (-514.2 – 796.8) | (-106.8 – 168.5) | (-12.18 – 8.438) |
| Living alone | -804.6*** | 164.3*** | -1,188*** | 174.7*** | -4.291*** |
|  | (-978.2 – -630.9) | (115.9 – 212.6) | (-1,366 – -1,010) | (136.6 – 212.8) | (-7.168 – -1.413) |
| Age group (ref. 18–45) |  |  |  |  |  |
| 46–65 | -2,023*** | -394.3** | -1,902*** | 93.48** | 30.10*** |
|  | (-2,899 – -1,146) | (-703.4 – -85.12) | (-2,843 – -960.8) | (18.90 – 168.1) | (23.17 – 37.04) |
| 66–85 | -3,129*** | -617.6*** | -3,063*** | 282.4*** | 62.08*** |
|  | (-3,997 – -2,261) | (-924.6 – -310.6) | (-3,996 – -2,131) | (208.0 – 356.8) | (55.25 – 68.92) |
| 86–106 | -5,023*** | -897.2*** | -5,151*** | 580.8*** | 97.77*** |
|  | (-5,914 – -4,133) | (-1,206 – -588.6) | (-6,103 – -4,200) | (468.5 – 693.1) | (89.02 – 106.5) |
| Cancer type (ref. lung cancer) | | | | | |
| Central nervous system | -1,371*** | 160.4* | -1,909*** | 325.4*** | 63.51*** |
|  | (-1,829 – -913.4) | (-10.13 – 330.9) | (-2,328 – -1,490) | (147.9 – 502.8) | (50.65 – 76.36) |
| Colorectal | 1,025*** | 23.68 | 1,023*** | 21.81 | -6.170*** |
|  | (757.0 – 1,294) | (-42.48 – 89.84) | (743.8 – 1,301) | (-33.45 – 77.06) | (-10.33 – -2.008) |
| Gastroenterological | 545.2*** | 109.2*** | 557.5*** | -96.25*** | -4.204** |
|  | (327.9 – 762.6) | (49.36 – 169.0) | (333.2 – 781.8) | (-137.8 – -54.72) | (-7.769 – -0.639) |
| Genitourinary | 169.0 | 12.61 | 278.2 | 21.03 | 11.50*** |
|  | (-191.3 – 529.3) | (-80.29 – 105.5) | (-96.72 – 653.1) | (-59.12 – 101.2) | (4.985 – 18.01) |
| Gynecological | 225.3 | 148.8** | 67.10 | -66.29* | -16.06*** |
|  | (-179.4 – 630.1) | (25.33 – 272.2) | (-341.7 – 475.9) | (-143.9 – 11.36) | (-22.26 – -9.846) |
| Head and neck | 550.6* | -34.76 | 439.2 | 171.2** | -0.682 |
|  | (-23.41 – 1,125) | (-167.1 – 97.62) | (-146.9 – 1,025) | (20.53 – 321.9) | (-10.01 – 8.649) |
| Hematology | 5,308*** | -293.7*** | 5,917*** | -123.4*** | -32.45*** |
|  | (4,791 – 5,824) | (-349.4 – -238.0) | (5,329 – 6,505) | (-185.6 – -61.28) | (-37.32 – -27.58) |
| Breast | -313.5 | -25.60 | -524.8*** | 164.4*** | 11.54*** |
|  | (-691.0 – 64.11) | (-128.3 – 77.07) | (-899.1 – -150.6) | (59.77 – 269.1) | (4.390 – 18.69) |
| Malignant melanoma | -497.7 | -28.51 | -412.0 | -140.7* | 17.46** |
|  | (-1,294 – 298.8) | (-234.9 – 177.9) | (-1,229 – 405.2) | (-281.7 – 0.362) | (1.288 – 33.63) |
| Other | 479.5*** | 39.79 | 586.9*** | -78.13** | -8.168*** |
|  | (140.1 – 818.9) | (-48.62 – 128.2) | (232.3 – 941.5) | (-141.5 – -14.74) | (-13.53 – -2.801) |
| Prostate | -346.4** | -121.2*** | -365.2** | 90.02** | 22.26*** |
|  | (-689.7 – -3.044) | (-198.4 – -44.07) | (-713.3 – -17.13) | (3.027 – 177.0) | (15.38 – 29.13) |
| Household income (ref. low) |  |  |  |  |  |
| Medium-low | 186.3 | 112.8*** | 166.3 | -5.793 | -0.808 |
|  | (-41.85 – 414.5) | (60.71 – 164.9) | (-69.93 – 402.5) | (-54.94 – 43.35) | (-4.565 – 2.948) |
| Medium-high | 65.32 | 122.9*** | 77.30 | -50.32** | -1.731 |
|  | (-171.7 – 302.4) | (69.09 – 176.7) | (-168.4 – 323.0) | (-99.77 – -0.865) | (-5.635 – 2.173) |
| High | 383.9*** | 247.5*** | 309.4** | -98.21*** | -7.401*** |
|  | (112.6 – 655.2) | (179.9 – 315.0) | (28.77 – 590.0) | (-151.3 – -45.08) | (-11.68 – -3.125) |
| Missing household income | -388.8** | 77.99* | -180.9 | -2.722 | 3.326 |
|  | (-744.0 – -33.49) | (-8.005 – 164.0) | (-553.9 – 192.0) | (-84.08 – 78.63) | (-2.939 – 9.590) |
| Children (ref. no children) |  |  |  |  |  |
| Has child under 18 | -305.9 | 113.8 | -470.7 | -95.60 | -13.36 |
|  | (-2,586 – 1,974) | (-550.8 – 778.4) | (-2,755 – 1,813) | (-538.3 – 347.1) | (-48.54 – 21.83) |
| Has child over 18 | 555.6*** | 69.77** | 533.8*** | -28.87 | 6.794*** |
|  | (310.0 – 801.2) | (11.09 – 128.5) | (280.6 – 787.1) | (-83.87 – 26.14) | (2.764 – 10.82) |
| Charlson Comorbidity | -12.12 | -34.16*** | -7.444 | 34.03*** | 3.272*** |
|  | (-93.07 – 68.84) | (-53.95 – -14.36) | (-91.56 – 76.67) | (16.96 – 51.10) | (1.937 – 4.607) |
| Time from death diagnosis until death | -1.425*** | 0.132*** | -1.901*** | 0.168*** | 0.0214*** |
|  | (-1.608 – -1.242) | (0.0791 – 0.185) | (-2.090 – -1.713) | (0.125 – 0.211) | (0.0182 – 0.0246) |
|  |  |  |  |  |  |
| Observations | 68,763 | 68,763 | 68,763 | 68,763 | 68,763 |
| Note: 95% confidence interval (95% CI), *** p<0.01, ** p<0.05, * p<0.1. Municipality of residence not displayed. | | | | | |

**Table A6.** Number of unmatched SPC patients and patients exposed and unexposed to timely discontinuation of CTT by each CEM iteration

|  | SPC exposed | SPC unexposed | Timely discontinuation of CTT exposed | Timely discontinuation of CTT unexposed |
| --- | --- | --- | --- | --- |
| Coarsened Exact Match iteration | Unmatched | Unmatched | Unmatched | Unmatched |
| CEM1 Age, cancer, sex, education, income, living alone, children, CCI, region (final) | 3,124 | 11,412 | 18,764 | 1,582 |
| CEM2 Age, cancer, sex, education, income, living alone, children, CCI | 919 | 3,515 | 6,110 | 401 |
| CEM3 Age, cancer, sex, education, income, living alone, children | 415 | 1,858 | 3,052 | 177 |
| CEM4 Age, cancer, sex, education, income, living alone | 204 | 710 | 1,305 | 70 |
| CEM5 Age, cancer, sex, education, household income | 104 | 295 | 494 | 17 |
| CEM6 Age, cancer, sex, education | 35 | 6 | 3 | 2 |
| CEM7 Age, cancer, sex | 35 | 0 | 0 | 0 |

- 1. CEM

In our preferred model, the patients were matched based on age (above or below 65 years) [50], sex, parental status, co-habitation status, cancer type, Charlson Comorbidity Index (CCI) score (under or above 1), socio-economic factors such as household income (in high, medium-high, medium-low, and low quantiles), level of education (high, medium, low), and region of residence. All the variables included were measured prior to treatment. We performed distinct matching procedures for SPC and for timely discontinuation of CTT. The results of these procedures provided matching weights for each individual, which were then utilized in the subsequent regression analysis.

**Table A7.** Marginal effect of exposure to SPC on expenditure (euro) according to CEM iterations gradually reducing the number of variables in the match

|  | Total | Hospital | Hospice | Community and home-based | Primary |
| --- | --- | --- | --- | --- | --- |
| CEM2 Cancer type, age, sex, education, income, living alone, children, CCI | | | | | |
| Exposed | €-3,114*** | €-4,315*** | €976.0*** | €163.0*** | €41.97*** |
|  | (-3,356 – -2,872) | (-4,545 – -4,086) | (944.5 – 1,008) | (63.15 – 262.8) | (39.21 – 44.73) |
| Observations | 62,772 | 62,772 | 62,772 | 62,772 | 62,772 |
| CEM3 Cancer type, age, sex, education, income, living alone, children | | | | | |
| Exposed | €-2,325*** | €-3,440*** | €850.7*** | €126.9** | €39.62*** |
|  | (-2,564 – -2,086) | (-3,674 – -3,205) | (821.3 – 880.0) | (26.56 – 227.2) | (37.07 – 42.18) |
|  |  |  |  |  |  |
| Observations | 64,897 | 64,897 | 64,897 | 64,897 | 64,897 |
| CEM4 Cancer type, age, sex, education, income, living alone | | | | | |
| Exposed | €-3,039*** | €-3,939*** | €833.8*** | €144.6*** | €38.00*** |
|  | (-3,284 – -2,793) | (-4,167 – -3,711) | (805.2 – 862.5) | (49.61 – 239.6) | (35.25 – 40.75) |
|  |  |  |  |  |  |
| Observations | 66,292 | 66,292 | 66,292 | 66,292 | 66,292 |
| CEM5 Cancer type, age, sex, education, income | | | | | |
| Exposed | €-3,187*** | €-4,339*** | €974.1*** | €137.2*** | €40.98*** |
|  | (-3,428 – -2,947) | (-4,567 – -4,111) | (942.8 – 1,005) | (41.71 – 232.7) | (38.31 – 43.65) |
|  |  |  |  |  |  |
| Observations | 66,807 | 66,807 | 66,807 | 66,807 | 66,807 |
| CEM6 Cancer type, age, sex, education | | | | | |
| Exposed | €-1,363*** | €-2,666*** | €758.3*** | €665.7*** | €48.57*** |
|  | (-1,655 – -1,072) | (-2,935 – -2,397) | (730.1 – 786.5) | (610.3 – 721.2) | (45.99 – 51.15) |
|  |  |  |  |  |  |
| Observations | 67,165 | 67,165 | 67,165 | 67,165 | 67,165 |
| CEM7 Cancer type, age, sex |  |  |  |  |  |
| Exposed | €-1,363*** | €-2,666*** | €758.3*** | €665.7*** | €48.57*** |
|  | (-1,655 – -1,072) | (-2,935 – -2,397) | (730.1 – 786.5) | (610.3 – 721.2) | (45.99 – 51.15) |
|  |  |  |  |  |  |
| Observations | 67,165 | 67,165 | 67,165 | 67,165 | 67,165 |
| Note: 95% confidence interval (95% CI), *** p<0.01, ** p<0.05, * p<0.1. | | | | | |

***Table A8.*** *Marginal effect of timely discontinuation of CTT on expenditure (euro) according to CEM iterations gradually reducing the number of variables in the match*

|  | Total | Hospice | Hospital | Community and home-based | Primary |
| --- | --- | --- | --- | --- | --- |
| CEM2 Cancer type, age, sex, education, income, living alone, children, CCI | | | | | |
| Exposed to timely discontinuation of CTT | €-3,643*** | €166.7*** | €-4,172*** | €163.4*** | €38.27*** |
|  | (-3,857 – -3,429) | (139.3 – 194.2) | (-4,409 – -3,936) | (134.4 – 192.4) | (35.84 – 40.71) |
|  |  |  |  |  |  |
| Observations | 82,598 | 82,598 | 82,598 | 82,598 | 82,598 |
| CEM3 Cancer type, age, sex, education, income, living alone, children | | | | | |
| Exposed to timely discontinuation of CTT | €-3,640*** | €158.7*** | €-4,174*** | €169.6*** | €38.00*** |
|  | (-3,853 – -3,427) | (131.3 – 186.0) | (-4,409–- -3,939) | (140.9 – 198.3) | (35.59 – 40.42) |
|  |  |  |  |  |  |
| Observations | 85,880 | 85,880 | 85,880 | 85,880 | 85,880 |
| CEM4 Cancer type, age, sex, education, income, living alone | | | | | |
| Exposed to timely discontinuation of CTT | €-3,604*** | €157.4*** | €-4,121*** | €177.1*** | €37.37*** |
|  | (-3,816 – -3,392) | (130.0 – 184.9) | (-4,355 – -3,887) | (148.6 – 205.5) | (34.97 – 39.78) |
|  |  |  |  |  |  |
| Observations | 87,734 | 87,734 | 87,734 | 87,734 | 87,734 |
| CEM5 Cancer type, age, sex, education, income | | | | | |
| Exposed to timely discontinuation of CTT | €-3,580*** | €157.7*** | €-4,059*** | €172.0*** | €38.20*** |
|  | (-3,791 – -3,369) | (130.4 – 185.1) | (-4,292 – -3,827) | (143.6 – 200.5) | (35.80 – 40.60) |
|  |  |  |  |  |  |
| Observations | 88,598 | 88,598 | 88,598 | 88,598 | 88,598 |
| CEM6 Cancer type, age, sex, education | | | | | |
| Exposed to timely discontinuation of CTT | €-3,540*** | €162.5*** | €-4,018*** | €174.4*** | €38.09*** |
|  | (-3,750 – -3,329) | (135.3 – 189.8) | (-4,251 – -3,786) | (146.1 – 202.8) | (35.69 – 40.49) |
|  |  |  |  |  |  |
| Observations | 89,104 | 89,104 | 89,104 | 89,104 | 89,104 |
| CEM7 Cancer type, age, sex |  |  |  |  |  |
| Exposed to timely discontinuation of CTT | €-3,561*** | €170.3*** | €-4,046*** | €146.4*** | €37.99*** |
|  | (-3,772 – -3,349) | (143.2 – 197.4) | (-4,279 – -3,813) | (121.1 – 171.7) | (35.59 – 40.40) |
|  |  |  |  |  |  |
| Observations | 89,109 | 89,109 | 89,109 | 89,109 | 89,109 |
| Note: 95% confidence interval (95% CI), *** p<0.01, ** p<0.05, * p<0.1. | | | | | |
